# Genetic depletion and pharmacological targeting of αv integrin in breast cancer cells impairs metastasis in zebrafish and mouse xenograft models

**DOI:** 10.1186/s13058-015-0537-8

**Published:** 2015-02-25

**Authors:** Yihao Li, Yvette Drabsch, Philippe Pujuguet, Jiang Ren, Theo van Laar, Long Zhang, Hans van Dam, Philippe Clément-Lacroix, Peter ten Dijke

**Affiliations:** Department of Molecular Cell Biology, Cancer Genomics Centre Netherlands and Centre for Biomedical Genetics, Leiden University Medical Center, Postbus 9600, 2300 RC Leiden, The Netherlands; Galapagos SASU, Avenue Gaston Roussel, 93230 Romainville, France; Ludwig Institute for Cancer Research, Science for Life Laboratory, Uppsala University, Box 595, 75124 Uppsala, Sweden; Life Sciences Institute, Zhejiang University, Hangzhou, Zhejiang 310058 China

## Abstract

**Introduction:**

Increased expression of αv integrins is frequently associated with tumor cell adhesion, migration, invasion and metastasis, and correlates with poor prognosis in breast cancer. However, the mechanism by which αv integrins can enhance breast cancer progression is still largely unclear. The effects of therapeutic targeting of αv integrins in breast cancer also have yet to be investigated.

**Methods:**

We knocked down αv integrin in MDA-MB-231 and MCF10A-M4 breast cancer cells, or treated these cells with the αv antagonist GLPG0187. The effects of αv integrin depletion on mesenchymal markers, transforming growth factor-β (TGF-β)/Smad signaling and TGF-β-induced target gene expression were analyzed in MDA-MB-231 cells by RNA analysis or Western blotting. The function of αv integrin on breast cancer cell migration was investigated by transwell assay *in vitro*, and its effect on breast cancer progression was assessed by both zebrafish and mouse xenografts *in vivo*. In the mouse model, GLPG0187 was administered separately, or in combination with the standard-of-care anti-resorptive agent zoledronate and the chemotherapeutic drug paclitaxel, to study the effects of combinational treatments on breast cancer metastasis.

**Results:**

Genetic interference and pharmacological targeting of αv integrin with GLPG0187 in different breast cancer cell lines inhibited invasion and metastasis in the zebrafish or mouse xenograft model. Depletion of αv integrin in MDA-MB-231 cells inhibited the expression of mesenchymal markers and the TGF-β/Smad response. TGF-β induced *αv integrin* mRNA expression and αv integrin was required for TGF-β-induced breast cancer cell migration. Moreover, treatment of MDA-MB-231 cells with non-peptide RGD antagonist GLPG0187 decreased TGF-β signaling. In the mouse xenografts GLPG0187 inhibited the progression of bone metastasis. Maximum efficacy of inhibition of bone metastasis was achieved when GLPG0187 was combined with the standard-of-care metastatic breast cancer treatments.

**Conclusion:**

These findings show that αv integrin is required for efficient TGF-β/Smad signaling and TGF-β-induced breast cancer cell migration, and for maintaining a mesenchymal phenotype of the breast cancer cells. Our results also provide evidence that targeting αv integrin could be an effective therapeutic approach for treatment of breast cancer tumors and/or metastases that overexpress αv integrin.

**Electronic supplementary material:**

The online version of this article (doi:10.1186/s13058-015-0537-8) contains supplementary material, which is available to authorized users.

## Introduction

Metastasis is a multi-step process in which cancer cells disseminate from the primary site to distant tissues or organs [1]. Breast tumors are commonly epithelial in origin, and their ability to invade is enhanced by modulators that stimulate epithelial-mesenchymal transition (EMT), such as transforming growth factor-β (TGF-β) and transcriptional repressors Snail, Slug, and Twist that are induced by TGF-β [2-4]. During the metastasis cascade, (epi)genetic changes in cancer cells and signals from the microenvironment promote EMT of the tumor cells *in situ,* which facilitates local invasion and intravasation into nearby tissues and circulation. Subsequently, circulating tumor cells with a mesenchymal morphology may extravasate out of the blood stream and invade secondary sites, which involves cell-matrix interactions [5]. Breast carcinoma cells are able to infiltrate into specific tissues, including bone, lung and brain. Within the new microenvironment the tumor cells start to proliferate, and develop into a macrometastatic lesion [6].

Integrins are cell-surface adhesion receptors consisting of α and β transmembrane protein subunits, which directly interact with extracellular matrix (ECM) components when regulating cell migration, proliferation, and cell survival via outside-inside and/or inside-outside signaling mechanisms [7]. In cancer, integrins contribute to tumor growth, invasion and metastasis [8]. One of the α integrins, αv, dimerizes with the β integrin subunits β1, β3, β5, β6 and β8, and has been implicated in the pathophysiology of malignant tumors [9]. Integrins αvβ3, αvβ5 and αvβ6 have been reported to be crucial for tumor cell adhesion, migration, survival, maintenance of stem cell phenotype and angiogenesis and for crosstalk with growth factors in the activation of oncogenes and inhibition of tumor suppressors [10-13].

αv integrin can be involved in activation of latent TGF-β by binding latency-associated peptide (LAP) [14], can interact with the TGF-β (type II) receptor and thereby promote TGF-β-induced responses in lung fibroblasts and mammary epithelial cells [15,16], and can interact with the TGF-β type III receptor endoglin and stimulate TGF-β/Smad signaling in endothelial cells [17]. Vice versa the TGF-β receptor can also mediate phosphorylation of certain β-chains of integrins and modulate their function in hepatocellular carcinoma [18]. Moreover, TGF-β can regulate αv integrin expression in breast epithelial cells and αv integrin can modulate TGF-β receptor expression in dermal fibroblasts [19,20]. Thus, αv integrin and TGF-β signaling show extensive interplay and αv integrin may be an effector and mediator of TGF-β signaling responses [21,22].

Human metastatic breast cancer cells residing in bone showed high αvβ3 integrin expression. The MDA-MB-231 subclone B02, established from bone metastases, was found to constitutively overexpress αvβ3 integrin compared to the parental MDA-MB-231 cells [23]. Although αv integrin seems to be an important pharmacological target to inhibit breast cancer metastasis, the mechanism by which it regulates metastatic breast cancer progression is largely unknown. In this study, selective knockdown of αv integrin expression or pharmacological inhibition of αv integrin function was found to potently mitigate the invasion and metastasis of breast cancer cells in zebrafish and mouse xenograft models. In line with previous studies in other cell types, mechanistic *in vitro* studies revealed an integrated interplay between αv integrin and TGF-β, a strong driver of invasion and metastasis of breast cancer. Moreover, maximum efficacy of bone metastasis inhibition in mice was accomplished when therapeutic targeting of αv integrin was combined with standard-of-care metastatic breast cancer treatments.

## Methods

### Cell culture and reagents

Human MDA-MB-231-luc cells [24] were obtained from Dr Clemens Löwik (Department of Radiology, Leiden University Medical Center, Leiden, The Netherlands) and Dr Gabri van der Pluijm (Department of Urology, Leiden University Medical Center, Leiden, The Netherlands). The MDA-231/B02-luc line was previously published [23] and used for mouse xenograft experiments. These MDA-MB-231 cell lines were maintained at 37°C in DMEM high glucose containing L-glutamine, 10% FBS and 100 U/ml Pen/Strep (Gibco, Invitrogen, Blijswijk, Netherlands). MCF10A-M4 cells were kindly provided by Dr Fred Miller (Barbara Ann Karmanos Cancer Institute, Detroit, USA) and maintained at 37°C in DMEM/F12 (Gibco, Invitrogen, Blijswijk, Netherlands) containing 5% horse serum (Gibco, Invitrogen, Blijswijk, Netherlands), 0.1 μg/ml cholera toxin (Merck Millipore, Amsterdam, Netherlands), 0.02 μg/ml epidermal growth factor (EGF), 0.5 μg/ml hydrocortisone (Sigma, Zwijndrecht, Netherlands), 10 μg/ml insulin (Sigma, Zwijndrecht, Netherlands), 50 μg/ml streptomycin, and 100 U/ml Pen/Strep (Gibco, Invitrogen, Blijswijk, Netherlands).

### Zebrafish embryo production and tumor cell injection

The transgenic zebrafish line Tg(fli1:GFP) was raised, staged and maintained according to standard procedures in compliance with the local Institutional Committee for Animal Welfare of the Leiden University Medical Center (LUMC). Tumor cell injection into zebrafish embryos was conducted as we previously described [24]. Briefly, approximately 400 fluorescent-labeled mammalian cells were injected into the duct of Cuvier (DoC). After implantation, zebrafish embryos (including non-implanted controls) were maintained at 33°C [25]. For each cell line or condition, data are representative of at least two independent experiments with at least 50 embryos per group.

### *In vivo* toxicity test of chemical compounds

GLP0187 (Galapagos NV, Mechelen, Belgium) was added to the zebrafish egg water 2 days post fertilization (dpf) for toxicity tests, or 2 days post implantation (dpi) for treatment, and refreshed every second day. For toxicity tests, embryo survival or malformation was scored daily. For GLPG0187 treatment, after 5 days embryos were fixed overnight in 4% buffered paraformaldehyde at 4°C. Embryos were placed in a glass-bottom 96-well plate (Greiner Bio One GmbH, Frickenhausen, Germany), and imaged as described.

### Lentiviral transduction

Lentivirus was produced by co-transfecting pLKO-1 (shRNA-knockdown) plasmids and helper plasmids pCMV-VSVG, pMDLg-RRE (gag/pol), and pRSV-REV into HEK293T cells. Cell supernatants were harvested 48 h after transfection and used to infect cells or stored at −80°C. pLKO-1 plasmids with specific shRNAs were obtained from Sigma, Zwijndrecht, Netherlands (MISSION® shRNA). We used TRCN-0000003240 and TRCN-0000010769 for αv integrin knockdown. For stable cell lines, cells were infected at 20% confluence for 24 h with lentiviral supernatants diluted 1:1 with normal culture medium in the presence of 8 μg/ml polybrene (Sigma, Zwijndrecht, Netherlands): 24 h after infection, cells were placed under puromycin (1 μg/ml) selection for 3 days or more.

### Fluorescence-activated cell sorting (FACS) analysis

Cells were washed once with PBS and harvested with 0.05% trypsin/0.025% EDTA. Detached cells were washed with EDTA, re-suspended in EDTA (4 × 10^4^/ml), and Fc-blocked for 15 minutes at room temperature. Anti-human αv integrin conjugated to Phycoerythrin (PE) (R&D systems, Abingdon, UK) was added and cells were incubated for 45 minutes at 4°C. PE-conjugated mouse IgG anti body (R&D systems, Abingdon, UK) was used as a negative control. Cells were washed three times in PBS and PE fluorescence was measured on a BD LSR II Cytometer. Results were analyzed with BD FACS Diva 6.1 software.

### Apoptosis assay

The apoptotic effects of αv integrin were examined using propidium iodide (PI) flow cytometry as previously described [26]. Detached and adherent cells were collected and labeled for 15 minutes at room temperature with PI (40 μg/ml) and immediately analyzed on a BD LSRII flow cytometer (BD Biosciences, Breda, Netherlands) using BD FACS Diva6.1 software.

### RNA isolation and real-time quantitative PCR (RT-PCR)

RNA was extracted with a NucleoSpin RNA II kit (BIOKE, Leiden, Netherlands) according to the supplier’s manual. For RT-PCR a RevertAid First Strand cDNA Synthesis Kit (Thermo Scientific, Leusden, Netherlands) was used. RT-PCR was performed on a CFX connect real-time PCR system (Bio-Rad, Veenendaal, Netherlands) and analyzed with CFX Manager software version 2.0 (Bio-Rad, Veenendaal, Netherlands). The sequences of the primers are given in the supplemental information. All samples were analyzed in triplicate and normalized to GAPDH.

The sequences of the primers were as follows: Integrin αv forward: 5′-CTTCTTGGTGGTCCTGGTAGG-3′; Integrin αv reverse: 5′-TTTCTGCCACTTGATCCGAAA-3′; GAPDH forward: 5′-AGCCACATCGCTCAGACA C-3′; GAPDH reverse: 5′-GCCCAATACGACCAAATC C-3′; N-cadherin forward: 5′-CAGACCGACCCAAACAGCAAC-3′; N-cadherin reverse: 5′-GCAGCAACAGTAAGGACAAACATC-3′;Snail forward: 5′-ACCACTATGCCGCGCTCTT-3′; Snail reverse: 5′-GGTCGTAGGGCTGCTGGAA-3′; Slug forward: 5′-ATGAGGAATCTGGCTGCTGT-3′; Slug reverse: 5′-GAGGAGAAAATGCCTTTGGA-3′; Vimentin forward: 5′-CCAAACTTTTCCTCCCTGAACC-3′; Vimentin reverse: 5′-CGTGATGCTGAGAAGTTTCGTTGA-3′; CTGF forward: 5′-TGCGAAGCTGACCTGGAAGAGAA-3′; CTGF reverse: 5′-AGCTCGGTATGTCTTCATGCTGGT-3′; IL-11 forward: 5′-ACTGCTGCTGCTGAAGACTC-3′; IL-11 reverse: 5′-CCACCCCTGCTCCTGAAATA-3′; PAI-1 forward: 5′- GCAGGACATCCGGGAGAGA-3′; PAI-1 reverse: 5′-CCTGAGAACCTCCCTTGACCTT-3′.

### Western blot analysis

Western blotting was carried out as previously described [27]. The primary antibodies used were anti-N-Cadherin (BD Biosciences, Breda, Netherlands #610920), anti-α-Smooth Muscle Actin (Sigma, Zwijndrecht, Netherlands #A2547), anti-Snail (Cell Signaling, Leiden, Netherlands #3879), anti-Smad2 (BD Biosciences, Breda, Netherlands #610842), anti-p-Smad2 (Cell Signaling, Leiden, Netherlands #3108), anti-Smad4 (Santa Cruz #sc7966), anti-TGFβRI (Santa Cruz, Heidelberg, Germany #sc 398), anti-TGFβRII (Santa Cruz, Heidelberg, Germany #sc-400), anti-Smad3 (Epitomics, Duiven, Netherlands #1735–1), anti-p-Smad3 (a kind gift from Dr Edward B Leof, Mayo Clinic, Rochester, Minnesota) and anti-β-actin (Sigma, Zwijndrecht, Netherlands #A5441). All the secondary antibodies were from Sigma, Zwijndrecht, Netherlands. Western quantification was performed using image J software.

### Transwell migration assay

Migration assays were performed in 24-well polyethylene terephthalate inserts (Corning Life Sciences, Amsterdam, Netherlands, 8.0- μm pore size): 1 × 10^4^ MDA-MB-231 cells were cultured in DMEM with 0.5% FBS and seeded in the upper compartment (replicas for each sample). Then the cells were treated with or without TGF-β3 (5 ng/ml) for 16 h, which allows cells to migrate to the lower side of the insert filter. Cells in the upper side of the filter membrane were removed with a cotton swab. Cells on the lower side of the filter were fixed in 4% paraformaldehyde, stained with crystal violet 0.5% and then counted and photographed by randomly choosing different views under the microscope.

### Animals and surgical procedures

Four- to five-week-old female Balb-c nu/nu mice (Charles River, les Oncins, France) were anesthetized with isofluorane and 5 × 10^5^ freshly harvested MDA-MB-231/B02 luc cells in 100 μl PBS were inoculated into the tail vein [28]. All mouse experiments were performed according to ethical guidelines edited by the Animal Institutional Care and Use Committee of Galapagos controlled by French Authorities (agreement number B 93 063 06, DDPP, Seine Saint Denis, France). The *Comité National de Rélexion Ethique sur l’Expérimentaion Animale* approved all the mice experiments for this study on 5 July 2012. All research using zebrafish, including housing and experiments, was carried out according to the international guidelines and approved by the local Institutional Committee for Animal Welfare (*Dier Ethische Commissie* (DEC) of the LUMC.

### Proliferation assay *in vitro*

MDA-MB-231 or MCF10A-M4 cells were seeded at 2,000 cells per well in a 24-well plate. GLPG 0187 treatment was started 12 h after seeding. The numbers of cells were counted each 36 or 24 h until 108 or 96 h after seeding. Each experiment was performed in triplicates and numbers were calculated with a T20 cell counter (Brio-Rad, Veenendaal, Netherlands).

### Proliferation assay in zebrafish

Approximately 70 mCherry-labeled MDA-MB-231 cells were injected in to the yolk sac of 2-dpf *fli1:GFP* Casper zebrafish embryos. Embryos were sorted 1 day post injection (dpi) by confocal microscopy to assess the fluorescent mass at the yolk sac and the images were scanned by z-stacks. Injected embryos were kept in 96-well plates at 33°C and scanned at 6 dpi. The relative volume of tumor cells was calculated by Stacks software.

### Immunoflurescence staining in zebrafish

Xenografted zebrafish embryos were fixed with 4% paraformaldehyde. Samples were first dehydrated with methanol followed by a rehydration step and treated with 10 ug/ml proteinase K for 10 minutes at 37°C. Then cells were blocked and permeabilized with 1% BSA and 0.5% Triton X-100 in PBS before incubation with primary antibody Ki 67 (Merck Millipore, Amsterdam, Netherlands #AB9260; 1:200 in blocking buffer) in 4°C for 12 h. Samples then were washed with 5% Triton X-100-PBS, incubated with donkey anti rabbit IgG Alexa Fluor 647 (Invitrogene #A31572 1:200 in blocking buffer) at room temperature for 2 h. After washing with PBS, the embryos were analyzed the using confocal microscope SP5 STED (Leica, Rijswijk, Netherlands).

### *In vivo* imaging and radiography

Metastatic tumor growth was followed weekly by bioluminescence imaging (BLI) with the NightOwl, (Berthold, Bad Wildbad, Germany). The BLI signal intensity was quantified as the sum of photons within a region of interest given as the total flux (photons/second).

### Statistical analysis

Statistical analysis was performed using Prism 4 software (GraphPad La Jolla, USA). Results are expressed as the mean ± SD. Two-way analysis of variance (ANOVA) followed by the two-tailed Student *t*-test were used. *P* <0.05 was considered to be statistically significant (*0.01 < *P* <0.05; **0.001 < *P* <0.01; *** *P* <0.001).

## Results

### Establishment of stable αv integrin knockdown in MDA-MB-231 cells

**Figure 1:**
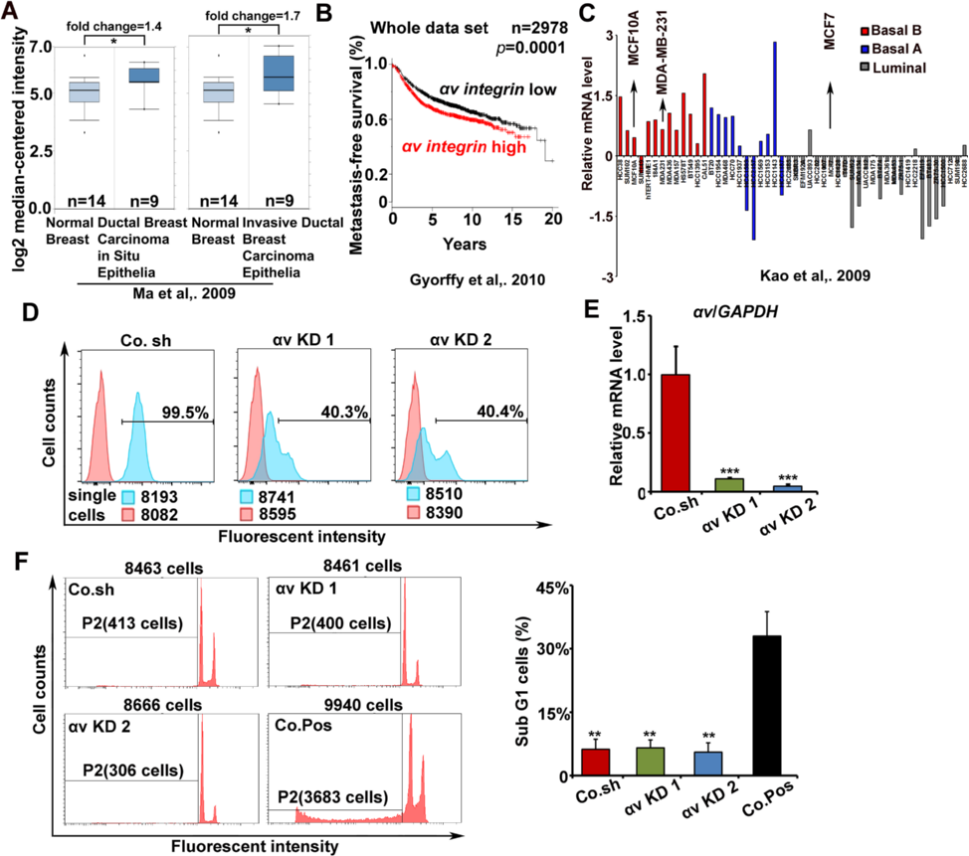
**Knockdown of αv integrin in breast cancer cells. (A)** Oncomine™ box plots of αv expression levels in normal breast and breast cancer data [29]. Normal versus ductal breast carcinoma *in situ* epithelia, *P* = 0.04; Normal versus invasive ductal breast carcinoma epithelia, *P* = 0.02. **(B)** Kaplan-Meier curves for the overall survival of breast carcinoma patients with high and low *αv integrin* expression; data obtained from the Kaplan-Meier plotter database [30]. *P*-values were calculated by log-rank test. **(C)** mRNA expression profile of *αv integrin* in breast cancer cell lines of different molecular subtypes (basal A, basal B and luminal) [31]. **(D)** FACS analysis of the αv integrin protein levels on the surface of control and αv integrin knockdown MDA-MB-231 cells, generated by stable infection with lentiviral vectors expressing two independent αv integrin targeting short hairpins (KD 1 and KD 2) or a control short hairpin (Co. sh). The percentage of αv^+^ cells was analyzed by flow cytometry. **(E)** Real-time quantitative PCR detection of *αv integrin* mRNA levels in control and αv integrin knockdown MDA-MB-231 cells. All samples were analyzed in triplicate and normalized to *GAPDH* (*P* <0.001). **(F)** Left, representative FACS profiles of propidium iodine (PI)-stained control MDA-MB-231 cells, αv knockdown cells and doxorubicin-treated MDA-MB-231 cells, a positive control (Co. Pos) for apoptosis detection. P2: subG1 cells. Right, percentage of sub G1 cells calculated by FACS. Co. Pos versus Co.sh or αv KD cells: *P* <0.01. Average ± SD of three independent experiments.

Analysis of Oncomine™ gene expression data [29] revealed that αv integrin is significantly upregulated in ductal breast carcinoma *in situ* epithelia or invasive ductal breast carcinoma epithelia (Figure 1A). Using publicly available databases we also found that *αv integrin* is highly expressed in breast cancer and that its expression was found to correlate with poor prognosis in 2,960 patients (Figure 1B) [30]. To further explore the expression of αv integrin in breast cancer cells, we analyzed *αv integrin* mRNA levels in breast cancer cell lines of different molecular subtypes by using a published dataset [31]. *Integrin αv* expression was higher in basal-like breast cancer cells, including MDA-MB-231 and MCF10A cell lines, than in luminal cell lines (Figure 1C). Moreover, the more aggressive MDA-MB-231 cells expressed more *αv integrin* than MCF10A-M4 (in short, M4) cells (Additional file 1: Figure S1A).

Next we performed loss of function analysis of αv integrin in basal-like breast cancer lines. Two independent stable MDA-MB-231 cell lines were generated in which αv integrin was knocked down using distinct shRNA targeting sequences. The knockdown efficiency was examined by FACS and Real-time qPCR analysis (Figure 1D and E). The cell surface protein and mRNA levels of αv integrin were significantly decreased upon shRNA-mediated depletion.

Previous studies showed that several integrins can affect cell survival [32]. We therefore tested whether αv integrin knockdown increased the amount of apoptotic MDA-MB-231 cells. After 7 days of infection, the percentage of sub G1 cells was 6% and 5% for the two specific (shRNAs), whereas MDA-MB-231 cells expressing the control shRNA contained 6% sub G1 cells. In contrast, cells treated with 1 μg/ml doxorubicin for 48 h as a positive control for apoptotic response contained 33% sub G1 cells (Figure 1F). Taken together, the shRNAs used efficiently target αv integrin in MDA-MB-231 cells and have no adverse effects on cell survival at the experimental conditions we investigated.

### αv integrin knockdown inhibits tumor invasion and metastasis in a zebrafish model

Next we investigated the role of αv integrin in breast cancer metastasis using a zebrafish xenograft model [33]. In a previous study it was reported that circulating cancer cells do not proliferate before colonization at the secondary site when examined in zebrafish embryos; phosphohistone-H3 postive tumor cells could only be detected after micro-metastasis formation. We analysed the effect of αv integrin knockdown on MDA-MB-231 proliferation in the primary xenotransplant site in embryonic zebrafish - the yolk sac. Additional file 1: Figure S1B shows the location of cells at 1 dpi, as well as the location (after migration) of cells at 6 dpi. The cells did not proliferate rapidly *in vivo* and the volume of shRNA control MDA-MB-231 cells is not significantly higher than the volume of αv integrin knockdown MDA-MB-231 cells at 6 dpi (Additional file 1: Figure S1C). To determine the percentage of breast cancer cells undergoing proliferation, we performed whole mount zebrafish immunostaining to examine Ki67 (a proliferation marker) expression in the transplanted cells (Additional file 1: Figure S1D). Both of the αv integrin knockdown cells and the control cells displayed approximately 65% proliferating cells. This suggests that knockdown of αv integrin does not significantly affect cell proliferation *in vivo* during the 6 day time period of the zebrafish xenograft assay.

**Figure 2:**
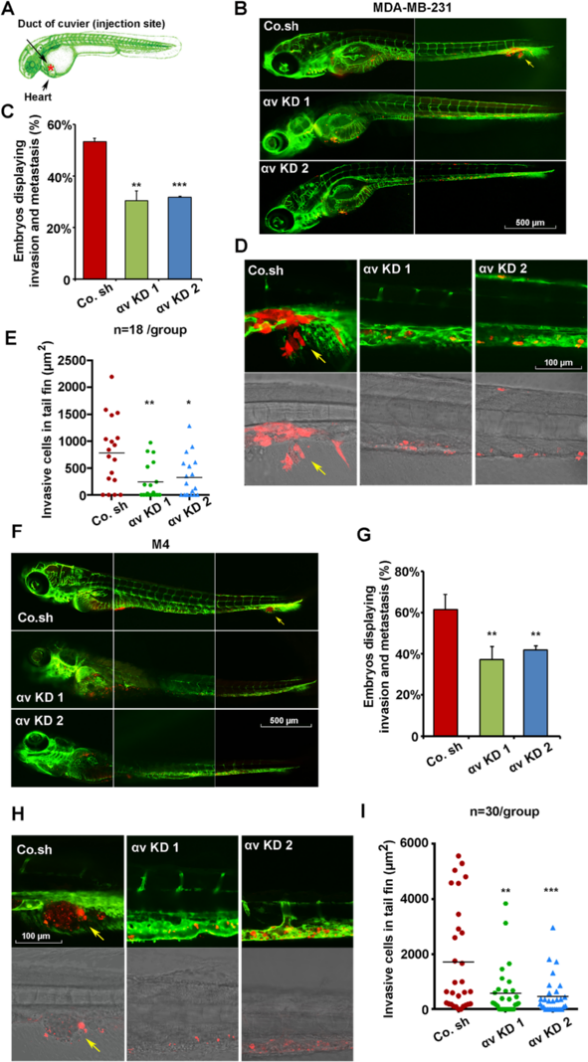
**Integrin αv is required for tumor cell invasion and metastasis in xenografted zebrafish. (A)** Schematic diagram of the zebrafish embryo xenograft model. **(B)** mCherry-labeled MDA-MB-231 cells infected with control shRNA (Co. sh) or αv integrin knockdown shRNAs (KD 1 and KD 2) were injected into zebrafish at 48 hours post fertilization (hpf). Confocal images were made 6 days post implantion (dpi). Arrows indicate invasive tumor cells, scale bar: 500 μm. **(C)** Percentage of embryos displaying invasion and metastasis detected at day 6 post injection. Data ± SD are representative of three independent experiments (each, n >50). Control versus αv KD 1 *P* <0.01; control versus αv KD 2 *P* <0.001. **(D)** High-resolution images of tumor cells in the posterior tail fin (upper panel, fluorescence; lower panel, transmitted). Arrows indicate invasive tumor cells, scale bar: 100 μm. **(E)** Area of invasive cells in each embryo (n = 18). Control versus αv KD 1 *P* <0.01; control versus αv integrin KD 2 *P* <0.05. **(F)** Invasion and metastasis in zebrafish xenografted with control or αv integrin knocked-down MCF10A-M4 cells at 6 dpi. Arrows indicate invasive tumor cells, scale bar: 500 μm. **(G)** Percentage of embryos displaying invasion and metastasis detected at day 6 post-injection. Control versus αv KD 1 *P* <0.01; control versus αv KD 2 *P* <0.01. **(H)** High-resolution images of tumor cells in the posterior tail fin (upper panel, fluorescence; lower panel, transmitted). Arrows indicate invasive tumor cells, scale bar: 100 μm. **(I)** Area of metastatic clusters in each embryo (n = 30). Control versus αv KD 1 *P* <0.01; control versus αv KD 2 *P* <0.001.

Zebrafish provide an important xenograft model for investigation of both tumor migration, angiogenesis and progression [34]. We previously showed that human breast cancer cells injected into the Duct of Cuvier (DoC) of zebrafish embryos will immediately disseminate throughout the circulatory system of the embryo, and during the next 6 days are capable of invading into the tail fin (3–30 cells), and forming micrometastasis (>30 cells) [35]. The fluorescently labeled αv integrin knockdown and control shRNA infected MDA-MB-231 cell lines were transplanted into the DoC of 48 hpf (hours post-fertilization) zebrafish embryos to study invasion and micrometastasis behavior *in vivo* (Figure 2A). We observed a dramatic decrease of invasion and micrometastasis in the MDA-MB-231 cells upon αv integrin knockdown (Figure 2B). Whereas control cells invaded into the avascular tail fin in 53% of zebrafish embryos, knockdown of αv integrin reduced the number of embryos displaying invasion to 30% with shRNA1, and 32% with shRNA2 (Figure 2C). Detailed pictures displayed the invading cells located into the collagen fibers of the tail fin in the control group, which is not seen in the αv integrin KD groups (Figure 2D). A significant reduction of the invasive area in each embryo was also detected in αv integrin KD groups (Figure 2E). Moreover, we consolidated these results with siRNA targeting αv integrin, which also reduced tumor invasion and micrometastasis in the zebrafish embryo (Additional file 2: Figure S2A, B and C).

To substantiate these results, we also examined the effect of αv integrin knockdown in MCF10A-M4 breast cancer cells. Stable αv integrin knockdown MCF10A-M4 cell lines were established by lentiviral shRNA infection (Additional file 3: Figure S3A-D). Unlike MDA-MB-231 cells, which migrate out as single cells into the avascular tail fin area, extravasated MCF10A-M4 cells invaded and formed a cluster of cells into the caudal hematopoietic tissue (CHT) between the dorsal aorta and caudal vein (Figure 2F, upper panel) [36]. Also, in this case, αv integrin knockdown significantly reduced breast cancer cell invasion and micrometastasis in the tail fin of zebrafish embryos (Figure 2F, lower panel). Although 61% of zebrafish embryos xenotransplanted with control shRNA expressing MCF10A-M4 cells displayed invasion/micrometastasis, upon αv integrin knockdown only 37% and 42% of the zebrafish embryos displayed invasion/micrometastasis (two independent shRNAs) (Figure 2G and H). Moreover, the invasive area of breast cancer cells was also decreased for the αv integrin KD cells (Figure 2I). These data indicate that αv integrin can promote breast cancer metastasis in multiple cell types and conditions.

### αv integrin is required for multiple mesenchymal features of MDA-MB-231 cells

**Figure 3:**
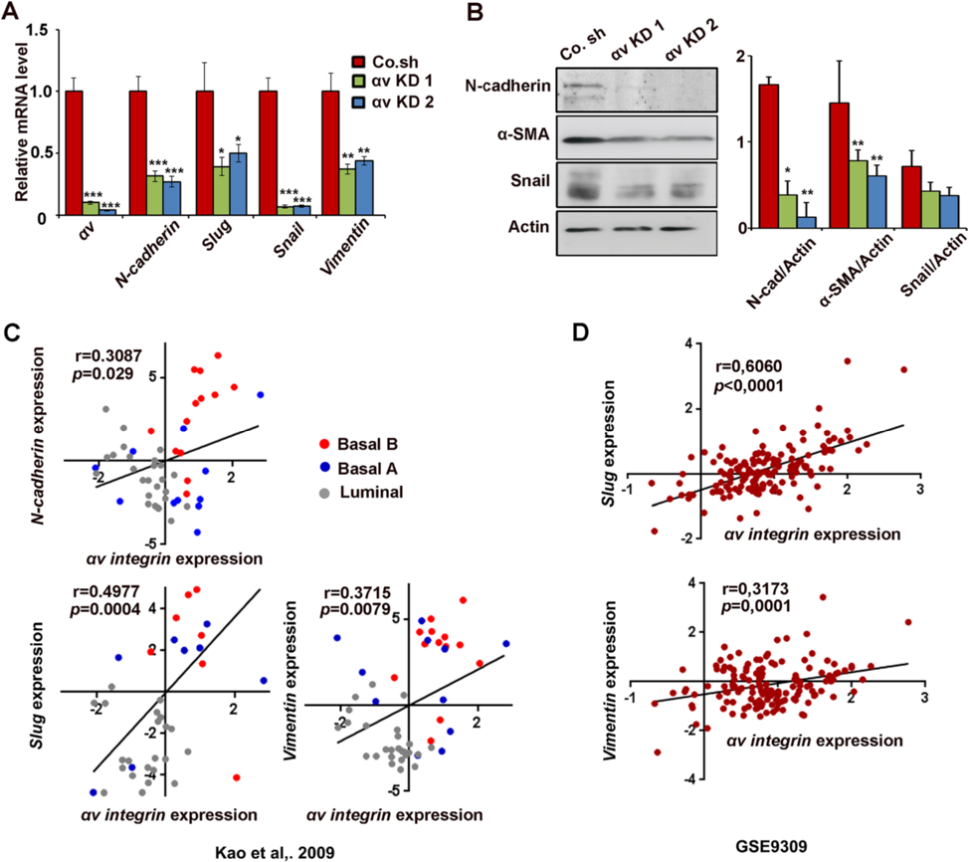
**Integrin αv regulates mesenchymal gene expression. (A)** Real-time qPCR mRNA analysis of various mesenchymal genes in MDA-MB-231 cells infected with control shRNA (Co. sh) or αv integrin knockdown shRNAs (KD 1 and KD 2). The RNA levels (± SD) of *αv integrin*, *N-Cadherin, Slug, Snail and Vimentin* are normalized to *GAPDH* expression. **(B)** Left, immunoblot detection of N-cadherin, Snail and α-smooth muscle actin protein in the cells shown in panel **A**. Right, quantification of the protein levels: three independent experiments, average ± SD. **(C)** *αv integrin* mRNA expression positively correlates with the mesenchymal markers *N-Cadherin*, *Slug* and *Vimentin* in a large breast cancer cell line dataset [31]. Pearsons’ coefficient tests were performed to assess statistical significance. **(D)** Scatter plots showing positive correlations between *αv integrin*, *Slug* and *Vimentin* expression in breast cancer patients using the Gene Expression Omnibus (GEO) [GEO:GSE9309]. Pearsons’ coefficient tests were performed to assess statistical significance.

The acquisition of mesenchymal properties is critical for early-stage carcinoma to switch to invasive malignancy, which is often associated with epithelial-mesenchymal transition (EMT), loss of epithelial differentiation and gain of a mesenchymal phenotype [37]. Therefore, we tested the effect of αv integrin on the expression of mesenchymal markers in MDA-MB-231 cells, which is a motile, highly aggressive and invasive cell line, and contains a typical mesenchymal morphology. mRNA analysis showed that knockdown of αv integrin resulted in a consistent decrease of *Snail*, *Slug*, *N-Cadherin*, and *Vimentin* mRNA levels (Figure 3A). Western blotting also showed significant reduction of N-cadherin, α-smooth muscle actin and Snail protein in αv integrin knockdown cells (Figure 3B). siRNA-mediated depletion of αv integrin caused reduced expression of mesenchymal markers as well (Additional file 4: Figure S4A). Knockdown of αv integrin in MCF10A-M4 also significantly decreased *Snail* expression (Additional file 4: Figure S4B). Depletion of αv integrin in MDA-MB-231 cells did not affect E-Cadherin protein levels (data not shown). These data indicate that in MDA-MB-231 cells αv integrin is required for the expression of various mesenchymal markers that are important for acquisition of an aggressive and metastatic phenotype.

By analyzing the mRNA expression profiles of a publicly available dataset of a panel of breast cancer cell lines, we found that high expression of *αv* integrin positively correlates with expression of the mesenchymal markers *Slug, N-Cadherin* and *Vimentin* (Figure 3C) [31]. Moreover, to investigate the relationship between αv integrin and mesenchymal marker expression in breast cancer patients, we analyzed the expression of *αv integrin*, *Slug* and *Vimentin* in the Gene Expression Omnibus (GEO) database [GEO:GSE9309] and observed that *αv integrin* expression positively has been found to correlate with *Slug* and *Vimentin* expression in 142 breast cancer patients (Figure 3D). Analysis of Oncomine™ gene expression data (TCGA database [38]) showed that both *αv integrin* and *Snail mRNA* are upregulated in invasive breast carcinoma (Additional file 4: Figure S4C). Together, these results illustrate that *αv integrin* expression correlates with mesenchymal characteristics in metastatic breast cancer cells.

### αv integrin-TGF-β interplay in MDA-MB-231 breast cancer cells

**Figure 4:**
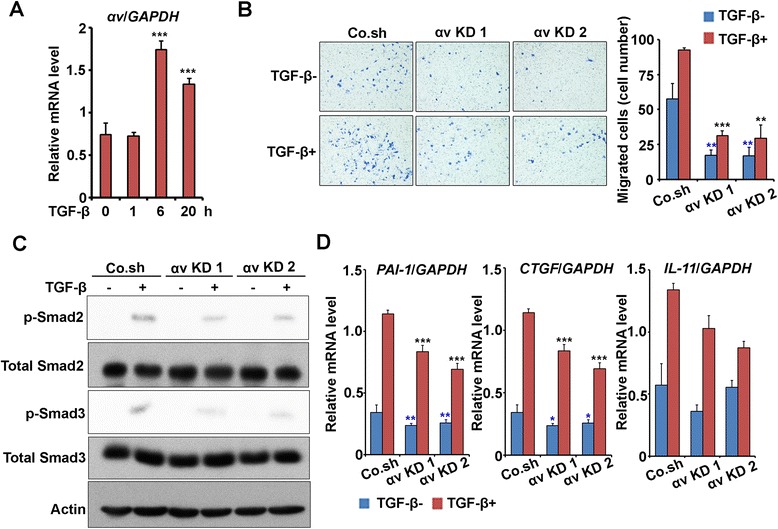
**αv integrin and transforming growth factor (TGF)-β signaling interaction in breast cancer progression. (A)** MDA-MB-231 cells were cultured with or without TGF-β for the indicated times, and RNA was isolated for *αv integrin* expression analysis by qRT-PCR. **(B)** Left, transwell assay. Control and knockdown MDA-MB-231 cells were treated with or without 5 ng/ml TGF-β for 16 h and migrated cells were stained. Right, average cell numbers of migrated cells ± SD. **(C)** Immunoblot analysis of phosphorylated Smad2 and Smad3 (p-Smad2 and 3) and total Smad2 and 3 in control or *αv* integrin knockdown MDA-MB-231 cells. Cells were treated with or without 5 ng/ml TGF-β for 2 h. **(D)** Knockdown of *αv* integrin in MDA-MB-231 cells inhibits expression of TGF-β target genes upon 6 h TGF-β treatment. mRNA levels of *PAI-1*, *CTGF*, and *IL-11* were assessed by real-time quantitative PCR, and normalized to *GAPDH* expression.

TGF-β plays a critical role during breast cancer metastasis, and also has been reported to stimulate αv integrin expression in certain cell types [15,19]. In addition, αv integrin may have an effector role in TGF-β-induced cell migration [19]. Therefore, we investigated the αv integrin-TGF-β interplay in MDA-MB-231 breast cancer cells. TGF-β treatment induced *αv integrin* mRNA expression at 6 and 20 h (Figure 4A), and depletion of αv integrin reduced both basal and TGF-β-induced MDA-MB-231 migration in a transwell assay (Figure 4B).

Next we investigated the role of αv integrin in TGF-β signaling using phosphorylation of its downstream effectors Smad2 and Smad3 as read-out. Upon knockdown of αv integrin in MDA-MB-231 cells, both TGF-β-induced Smad2 and Smad3 phosphorylation (as well as TGF-β-induced p38 MAP kinase) were significantly inhibited (Figure 4C and Additional file 5: Figure S5A). The total levels of Smad2, Smad3, Smad4 and TGFβ receptors remained unaltered (Additional file 5: Figure S5B). Moreover, the TGF-β-induced Smad3/Smad4-driven transcriptional response was mitigated upon depletion of αv integrin in 293 T cells (Additional file 5: Figure S5C). These results are consistent with previous data obtained in glioblastoma cells [39]. Subsequently, the effect of αv integrin depletion on TGF-β- induced target gene expression in MDA-MB-231 cells was analyzed. Figure 4D shows that TGF-β induction of *PAI-1*, *CTGF* and *IL-11* was significantly decreased in the αv integrin knockdown cells. In line with the reduction in basal phosphorylation of Smad2 and Smad3, the basal expression of *PAI-1* and *CTGF* was also affected upon αv integrin knockdown (Figure 4D). Collectively, these results revealed that αv integrin is a target of TGF-β, needed for efficient TGF-β/Smad signaling, and required for TGF-β-induced cell migration.

### Therapeutic targeting of αv integrin by GLPG0187 antagonises breast cancer metastasis in a zebrafish model

**Figure 5:**
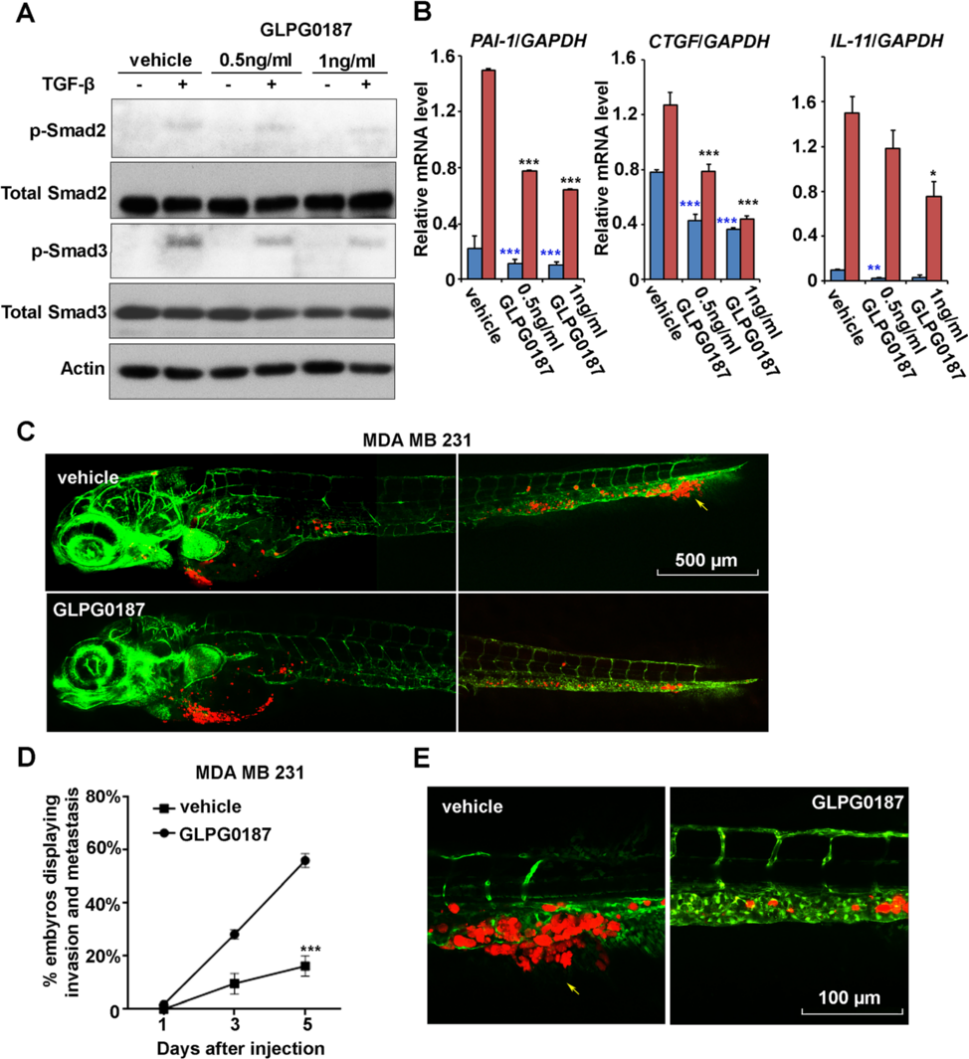
**GLPG0187 suppresses breast tumor invasion and metastasis in zebrafish. (A)** MDA-MB-231 cells were treated with vehicle, 0.5 ng/ml or 1 ng/ml of GLPG0187 for 48 h with or without 5 ng/ml transforming growth factor (TGF)-β for 2 h and immunoblotted as shown. **(B)** MDA-MB-231 cells were treated with vehicle, 0.5 ng/ml or 1 ng/ml of GLPG0187 for 48 h with or without 5 ng/ml TGF-β for 6 h and subjected to quantitative PCR analysis. mRNA levels of *PAI-1*, *CTGF*, and *IL-11* were normalized to *GAPDH* expression. **(C)** MDA-MB-231 cells were pretreated for 6 h with 1 ng/ml GLPG0187 or vehicle, then injected into the blood circulation of zebrafish 48-h post fertilization and followed with or without 0.5 ng/ml GLPG0187 treatment, as indicated. Confocal images of overview zebrafish were made 5 days post implantion (dpi). Arrows indicate invasive tumor cells, scale bar: 500 μm. **(D)** Percentage of embryos displaying invasion and metastasis at 1, 3, and 5 dpi is shown; vehicle versus GLPG0187 *P* <0.001. **(E)** Representative images of the zebrafish described in panel **D**. Experimental micrometastasis was detected in the posterior tail fin at 5 dpi. Arrows indicate invasive tumor cells, scale bar: 100 μm.

GLPG0187 is a recently developed non-peptide RGD antagonist, which inhibits αv integrin-ligand interactions [19,40]. More specifically, this small molecule compound was found to exhibit high affinity for αvβ1, αvβ3, αvβ5, αvβ6, αvβ8 as well as α5β1 in *in vitro* competitive binding assays [19]. The impact of GLPG0187 on cell proliferation/viability was evaluated by cell counting. Treatment of MDA-MB-231 cells with 0.5 or 1 ng/ml GLPG0187 did not change the rate of proliferation significantly (Additional file 5: Figure S5D). Next we examined whether GLPG0187 affects TGF-β signaling in MDA-MB-231 cells. GLPG0187 treatment attenuated TGF-β-induced phosphorylation of Smad2 and Smad3 in a dose-dependent manner (Figure 5A). In addition, we observed that GLPG0187 dose-dependently inhibited TGF-β-induced *PAI-1*, *CTGF* and *IL-11* mRNA expression in MDA-MB-231 cells (Figure 5B). Taken together these results indicate that αv integrin-inhibition by GLPG0187, as with genetic depletion of αv integrin mitigates TGF-β signaling.

To test the potential therapeutic advantage of αv integrin targeting, MDA-MB-231 breast cancer cells were pretreated for 6 h with 1 ng/ml GLPG0187. Cells were subsequently xenotransplated into 48-hpf *Fli*:GFP zebrafish embryos, and 0.5 ng/ml GLPG0187 (a dose at which no toxicity was observed in zebrafish embryos) was added to the water every second day after injection. As a result, the embryos transplanted with MDA-MB-231 cells treated with the vehicle developed an aggressive tumor lesion on the tail fin in 56% of the embryos. In contrast, pretreatment of MDA-MB 231 cells with GLPG0187 (1 ng/ml) for 6 h before injection and treatment of zebrafish embryos with GLPG0187 (0.5 ng/ml) after injection caused a significant reduction of tumor cell invasion (16%) (Figure 5C,D and E). GLPG0187 also inhibited the invasion of MCF10A-M4 cells in zebrafish embryos (data not shown). These data show that GLPG0187 blocks breast cancer invasion, and subsequent micrometastasis in this zebrafish model.

### GLPG0187 inhibits progression of established bone metastasis

**Figure 6:**
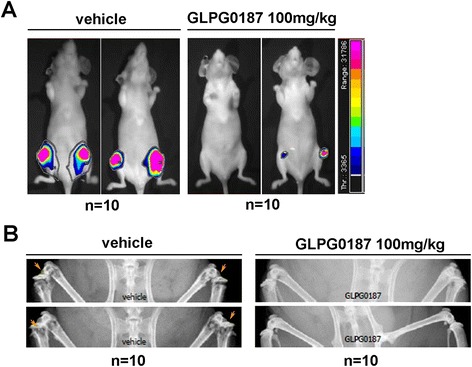
**GLPG0187 inhibits progression of established bone metastasis. (A)** Bioluminescent imaging at week 6 of two representative mice injected with MDA-MB-231 cells and administered with vehicle or GLPG0187 (100 mg/kg) weekly. **(B)** Representative radiographic images illustrating GLPG0187 (100 mg/kg) efficacy on associated osteolytic lesions in this mouse model of human breast cancer bone metastasis.

According to the previous zebrafish results, GLPG0187 shows potent anti-tumor progression activities. A phase-I trial in healthy volunteers with GLPG0187 has started (ClinicalTrials.gov Identifier: NCT01313598, NCT01580644, NCT00928343). To further confirm anti-metastatic activity of GLPG0187 we performed xenograft experiments in a mouse model of female nude mice that were intravenously injected with MDA-MB-231/BO2 cells, a subclone of MDA-MB-231 that stably expresses luciferase, and which selectively metastasizes to bone [23]. First detection of metastatic cells in bone by luminescence was possible at day 13, which allowed randomization. At the day following randomization, mice were treated with vehicle or GLPG0187 weekly to evaluate whether GLPG0187 can inhibit established metastases. Forty-two days after injection, mice treated only with vehicle displayed aggressive bone metastases that developed continuously, whereas mice treated with GLPG0187 only developed tiny detectable metastases in bone (Figure 6A). Compared with the vehicle-only group, significantly reduced bone lesions were observed when treated with GLPG0187 42 days after injection (Figure 6B). These results suggest that GLPG0187 reduces the osteolytic bone-metastatic ability of MDA-MB-231 cells.

### GLPG0187 achieves maximum efficacy when combined with standard-of-care metastatic breast cancer treatments

**Figure 7:**
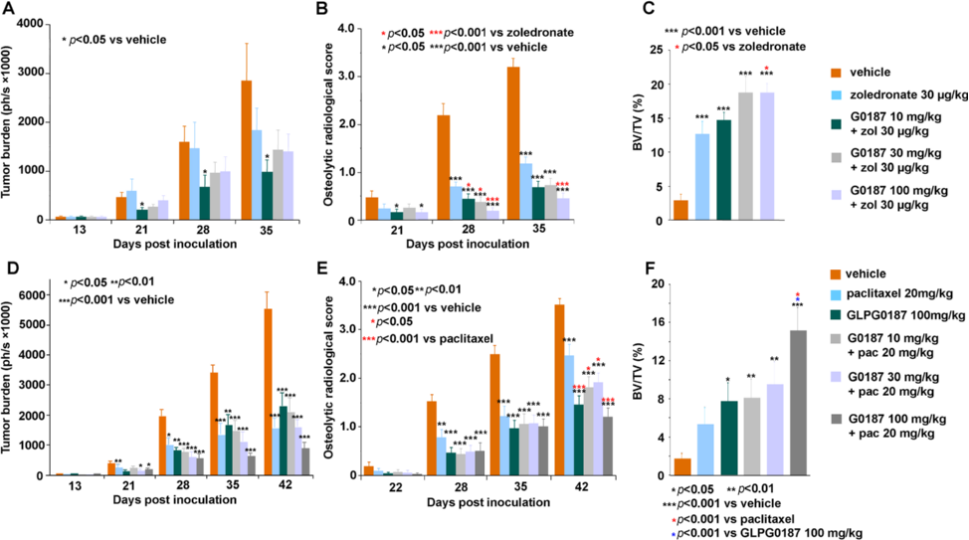
**High efficacy in combination therapy of GLPG0187 and standard-of-care treatments. (A-C)** GLPG0187 combined with the antiresorptive standard-of-care agent zoledronate in the MDA-MB-231-mouse bone metastasis model (Figure 6) shows efficacy on the tumor burden, tumor-induced osteolysis, and tibiae bone volume/trabecular bone volume (BV/TV) parameter measured by micro computed tomography (Μct). **(D-F)** GLPG0187 combined with the chemotherapeutic standard-of-care agent paclitaxel in the bone metastasis model shows efficacy on the tumor burden, tumor-induced osteolysis, and tibiae BV/TV parameter measured by μCT. Relevant doses of standard-of-care therapeutic agents were administered: zoledronate (30 μg/kg weekly subcutaneaously) , paclitaxel (20 mg/kg weekly intraperitoneally). GLPG0187 was tested at 10, 30 and 100 mg/kg, orally, twice daily either separately or in combination with the above therapeutic agents.

Next we studied the anti-metastatic activities of GLPG0187 in combination with standard-of-care treatments for metastatic breast cancer with the aim of checking the potential antagonistic effects of these treatments in a mouse model. The results showed that GLPG0187 treatment exerted an anti-metastatic effect when MDA-MB-231 cells metastasized to bone. Importantly, combinations of GLPG0187 with the antiresorptive agent zoledronate showed superior activity on both tumor burden and the associated osteolytic lesions compared to each treatment separately (Figure 7A and B). We observed up to 84% inhibition of the tumor burden and full bone protection against osteolysis, as measured by bone volume, compared to intact control mice (Figure 7C). When GLPG0187 was combined with the chemotherapeutic drug paclitaxel, the formation of the tumor burden and tumor-induced osteolysis were dramatically decreased in comparison with the other treatment (Figure 7D and E). Also, a diminished tumor burden in tibiae was achieved with combination treatment (Figure 7F). In addition to the absence of an antagonistic effect, the combination of GLPG0187 with approved antiresorptive or chemotherapeutic agents shows superior efficacy when compared to each treatment taken separately.

## Discussion

Studies correlating integrin expression levels in human tumors with pathological outcome, such as patient survival and metastasis have identified several integrins that might have an important role in cancer progression. Here we investigated the role of αv integrin in breast cancer invasion and metastasis by using preclinical models for human breast cancer. Selective shRNA-mediated knockdown of αv integrin expression was found to potently mitigate invasion and metastasis of breast cancer cells in zebrafish and mouse xenograft models. This study further established the zebrafish embryo xenograft model as a robust and dependable animal model for cancer research. Transplanted fluorescently labeled mammalian tumor cells in zebrafish can survive, invade and metastasize, and thus display similar behavior to cells transplanted in the traditional mammalian models [41]. Moreover, the zebrafish embryo model also enables us to monitor the metastasis cascade at the single-cell level.

Metastasis is associated with acquisition of mesenchymal characteristics. Here we explored the impact of αv integrin on maintenance of mesenchymal morphology in mammary carcinoma. Reduced expression of αv integrin in MDA-MB-231 cells was associated with downregulation of mesenchymal effectors. Moreover, αv integrin was upregulated by TGF-β and found to participate in stimulation of TGF-β-induced cell migration, TGF-β-target gene activation, and TGF-β/Smad signaling. This is in line with previous studies showing that various integrins can stimulate TGF-β-induced signaling at multiple levels [14,21,22,42,43].

Whether the reduction in basal migration, basal phosphorylation of Smad2 and basal expression of *PAI1* and *CTGF* after αv integrin knockdown is caused by reduction of integrin-mediated activation of latent TGF-β, or by interference with autocrine active TGF-β-like signaling remains to be established. Irrespective of the exact mechanism, our results indicate that αv integrin at least in part mediates TGF-β/Smad signaling, which has been shown to be critical for the bone metastasis signature of MDA-MB-231 cells [44,45]. This notion is consistent with previous observations that interfering with TGF-β signaling inhibits integrin expression and TGF-β-induced metastasis of breast cancer cells [46], and that expression of αvβ3 integrin is a key determinant for homing of breast cancer cells to bone [47]. In addition, αv integrin will also have functions that are independent of TGF-β, such as outside-in and inside-out signaling, which may also be important for invasive and metastatic properties of breast cancer cells [21]. In the bone metastatic lesions, integrins are essential for the interaction between tumor cells and ECM, and also play a role in osteoclast-bone binding [48]. For example, αvβ3 has high affinity binding with the bone marrow stroma ligands, osteopontin and vitronectin, to promote prostate bone metastasis [49].

Interestingly, analysis of a previously published dataset, in which 52 breast cancer cell lines were transcriptionally profiled [31], revealed significant correlation between expression of α*v integrin* and the mesenchymal markers *N-Cadherin*, *Slug* and *Vimentin*. Moreover, *αv integrin* was found to be highly expressed in mesenchymal breast cancer, with an invasive and metastatic phenotype. A previous study revealed that BMP7 may inhibit TGF-β-induced EMT and bone metastasis in both breast and prostate cancer by decreasing αvβ3 integrin expression [19,50]. Moreover, antibody-mediated blockade of αv integrin function, in particular of αvβ6, downregulates TGF-β-induced EMT and inflammation associated with fibrosis, metastasis and cancer [51-54].

Integrin-mediated signaling can enhance cell survival through several mechanisms. These include regulation of the expression of BCL-2 [55], FLIP [56], or survival-promoting pathways such as PI3K-AKT [57] or nuclear factor κB (NF-κB) signaling [58]. Our findings showed that αv integrin knockdown does not significantly enhance apoptosis in the two breast cancer cell lines studied. This might be explained by the fact that we were targeting a single integrin, and did not fully disrupt integrin-mediated cell survival.

Integrins are appealing therapeutic targets because they are expressed in various cell types involved in tumor progression, and interact with growth factor receptors. Several preclinical studies have shown that integrin antagonists inhibit tumor growth by affecting both tumor cells and tumor-associated host cells (that is, the angiogenic endothelium). Here we assessed the effect of a new non-peptide integrin-specific antagonist, GLPG0187, on breast cancer progression. Administration of GLPG0187 resulted in a decrease of tumor invasion in the zebrafish embryo model. Furthermore, GLPG0187 effectively inhibited the progression of established bone metastasis in a mouse model of breast cancer and showed superior activity when combined with antiresorptive and chemotherapeutic standard-of-care agents. It should be noted that genetic depletion and pharmacological inhibition represent two different approaches to inhibit αv integrin function.

Demonstrating that GLPG0187 inhibits bone metastasis in mice is not sufficient to conclude that the αv integrins on tumor cells are targeted and responsible for the observed effect. This inhibitor acts in a non cell-autonomous manner and also selectively inhibits the interaction of αv integrins of non-tumor cells, for example, stromal cells with extracellular matrix components [19]. Additional studies in which, for example, the effect of genetic depletion of αv integrin in tumor cells on bone metastasis is analyzed, are needed to directly implicate αv integrin effects on tumor cells in the bone metastatic response. The (dose-dependent) selectivity spectra of shRNA-mediated depletion of the total protein and pharmacological inhibition with GLPG0187 can also be different. For instance, responses that are initiated upon αv integrin activation might be subject to different thresholds of signaling intensity/duration. Functions of αv integrin that are not dependent on its interaction with the ECM may not be affected by GLPG0187, but will be affected by depletion of the protein. Therefore, these two different approaches to address the functional role of αv integrin in breast cancer cells complement and strengthen each other.

## Conclusions

Here we demonstrate that αv integrin is required for breast cancer cell invasion and metastasis by regulating mesenchymal markers expression and crosstalk with TGF-β signaling. We translate findings obtained in cell culture and an innovative cost effective and rapid zebrafish xenograft model to an *in vivo* mouse model. More specifically, the αv integrin small molecule antagonist GLPG0187 inhibited bone metastasis, and maximum efficacy was achieved when combined with antiresorptive zoledronate or chemotherapeutic paclitaxel. An important issue concerning the interpretation of efficacy of GLPG0187 is that with this approach we cannot exclude possible effects through targeting of integrins on other cell types than the tumor cells, including cells in the metastatic niche or in the vasculature [19]. In bone metastasis the activity of osteoclasts is important for bone resorption, a process that can be inhibited by targeting integrins. Various integrins, including αvβ3, have been implicated in tumor angiogenesis. Taken together, our data suggest that breast cancer patients with high levels of αv integrin will most likely benefit from a combinatorial pharmacological approach that includes αv integrin targeting.

## Additional files

Additional file 1: Figure S1. Proliferation of αv integrin deficient MDA-MB-231 cells in zebrafish. **(A)** qPCR analysis of MDA-MB-231 cells and MCF10A-M4 cells. mRNA levels of *αv* were normalized to *GAPDH* expression. **(B)** Embryos were injected with control shRNA or αv integrin knockdown MDA-MB-231 cells and examined by fluorescence microscopy at 1 and 6 days post implantation (dpi) (one representative of 15 embryos is shown; scale bar: 200 μm). **(C)** Relative average tumor cell volume at 1 and 6 dpi. **(D)** Ki67 positive proliferating breast tumor cells at the primary xenografted site at 6 dpi. Green: *Fli-1* GFP, blue: Ki67, red: mCherry-MDA-MB-231 cells, scale bar: 100 μm. Additional file 2: Figure S2. Inhibition of tumor progression in zebrafish by siRNA-mediated knockdown of αv integrin. **(A)** *fli1:GFP* Casper zebrafish were injected with mCherry-labeled MDA-MB-231 cells transfected with control siRNA or αv integrin knockdown siRNA. Confocal images were photographed at 6 days post implantation (dpi). Arrows indicate invasive tumor cells, scale bar: 500 μm. **(B)** Percentage of embryos displaying invasion and metastasis at day 6 post-injection. Data are representative of two independent experiments (each, n >50). C. High-resolution images of the posterior tail to visualize single metastatic tumor cells (upper panel, fluorescence; lower panel, transmitted). Arrows indicate invasive tumor cells, scale bar: 100 μm. Additional file 3: Figure S3. Stable knockdown of αv integrin in MCF10A-M4 cells. **(A)** Representative images of MCF10A-M4 cells infected with lentivirus expressing control-shRNA or αv integrin knockdown shRNAs. **(B)** mRNA level of *αv integrin* in control and αv integrin knockdown MCF10A-M4 cells detected by quantitative PCR. **(C)** Representative Fluorescence-activated cell sorting (FACS) profiles of propidium iodine-stained control M4 cells, αv integrin KD 1 and 2 MCF10A-M4 cells and as positive apoptotic control doxorubicin treated MCF10A-M4 cells (Co.Pos). P2: subG1 cells. **(D)** Percentage of sub G1 cells calculated by FACS. Average ± standard deviation of 3 independent experiments). Additional file 4: Figure S4. Effects of αv integrin on mesenchymal markers. A. mRNA expression of mesenchymal makers in control siRNA or αv integrin siRNA transfected MDA-MB-231 cells. The mRNA levels of *αv integrin, N-Cadherin, Slug, Snail* and *Vimentin* are normalized to *GAPDH* expression. *P*-values were calculated by the two-sided Student *t*-test. **(B)** Quantitative PCR analysis of Snail mRNA in control and αv integrin knockdown MDA-MB-231 cells. The RNA levels (± SD) of *Snail* are normalized to *GAPDH* expression. **(C)** Oncomine™ box plots of *αv integrin* and *Snail* expression levels in normal breast and invasive breast carcinoma [38]. Additional file 5: Figure S5. Effect of genetic depletion of αv integrin on expression of transforming growth factor (TGF)-β pathway components and effect of GLPG0187 on cell proliferation/viability in MDA-MB-231 cells. **(A)** Western blot analysis of total and phosphorylated p38 in control and αv integrin knockdown MDA-MB-231 cells with or without 5 ng/ml TGF-β for 2 h. **(B)** Immunoblot analysis of phosphorylated Smad2 and 3, total Smad2 and 3, Smad4, TGF-β receptors in MDA-MB-231 αv integrin knockdown cells. **(C)** Analysis of the TGF-β-induced Smad3 transcriptional response: 293 T cells were transfected with the CAGA12-Luc transcriptional reporter, control siRNA or αv siRNA. Cells were treated with or without 5 ng/ml TGF-β for 16 h. **(D)** Proliferation curves of MDA-MB-231 cells upon treatment with vehicle, 0.5, 1 or 10 ng/ml GLPG0187 for 36, 72 and 108 h.

## Abbreviations

BLI: bioluminescence imaging
BSA: bovine serum albumin
CTGF: connective tissue growth factor
DMEM: Dulbecco's modified Eagle's medium
DoC: duct of Cuvier
dpf: days post-fertilization
dpi: days post implantation
ECM: extracellular matrix
EMT: epithelial-mesenchymal transition
FACS: fluorescence-activated cell sorting
FBS: fetal bovine serum
hpf: hours post fertilization.
IL-11: interleukin 11
integrin: αv vitronectin receptor subunit α
LUMC: Leiden University Medical Center
NF-κB: nuclear factor κB
PAI-1: plasminogen activator inhibitor-1
PBS: phosphate-buffered saline
PE: phycoerythrin
PI: propidium iodide
RT-PCR: real-time quantitative PCR
TGF-β: transforming growth factor-β
