# Supplementary figures and images for: Genetic depletion and pharmacological targeting of αv integrin in breast cancer cells impairs metastasis in zebrafish and mouse xenograft models
Source: Breast Cancer Res. 2015 Feb 25;17(1):28. doi: 10.1186/s13058-015-0537-8 (PMC4381510; doi:10.1186/s13058-015-0537-8)

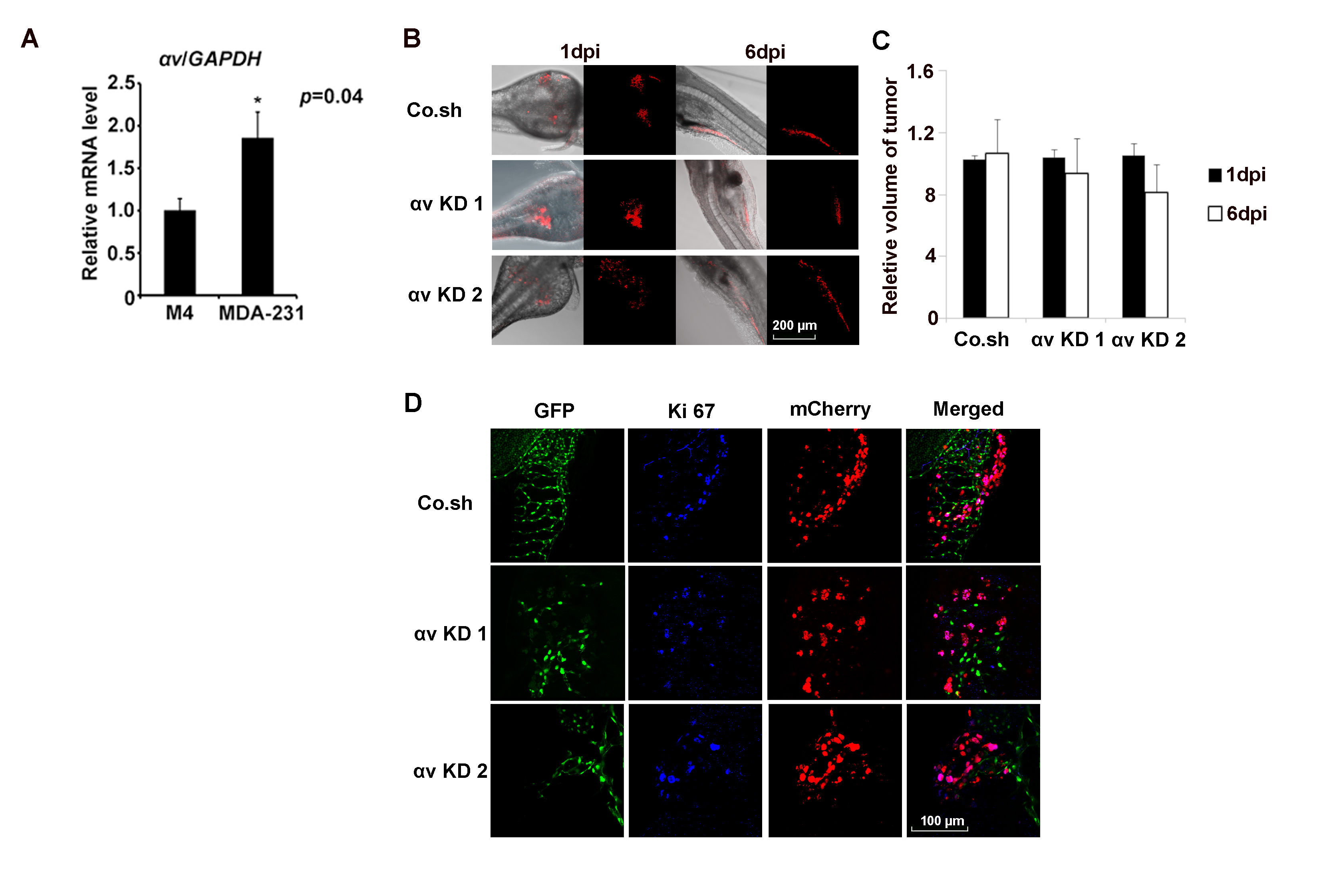

Supplement: Additional file 1: Figure S1. — Proliferation of αv integrin deficient MDA-MB-231 cells in zebrafish. (A) qPCR analysis of MDA-MB-231 cells and MCF10A-M4 cells. mRNA levels of αv were normalized to GAPDH expression. (B) Embryos were injected with control shRNA or αv integrin knockdown MDA-MB-231 cells and examined by fluorescence microscopy at 1 and 6 days post implantation (dpi) (one representative of 15 embryos is shown; scale bar: 200 μm). (C) Relative average tumor cell volume at 1 and 6 dpi. (D) Ki67 positive proliferating breast tumor cells at the primary xenografted site at 6 dpi. Green: Fli-1 GFP, blue: Ki67, red: mCherry-MDA-MB-231 cells, scale bar: 100 μm. [file 13058_2015_537_MOESM1_ESM.jpeg]

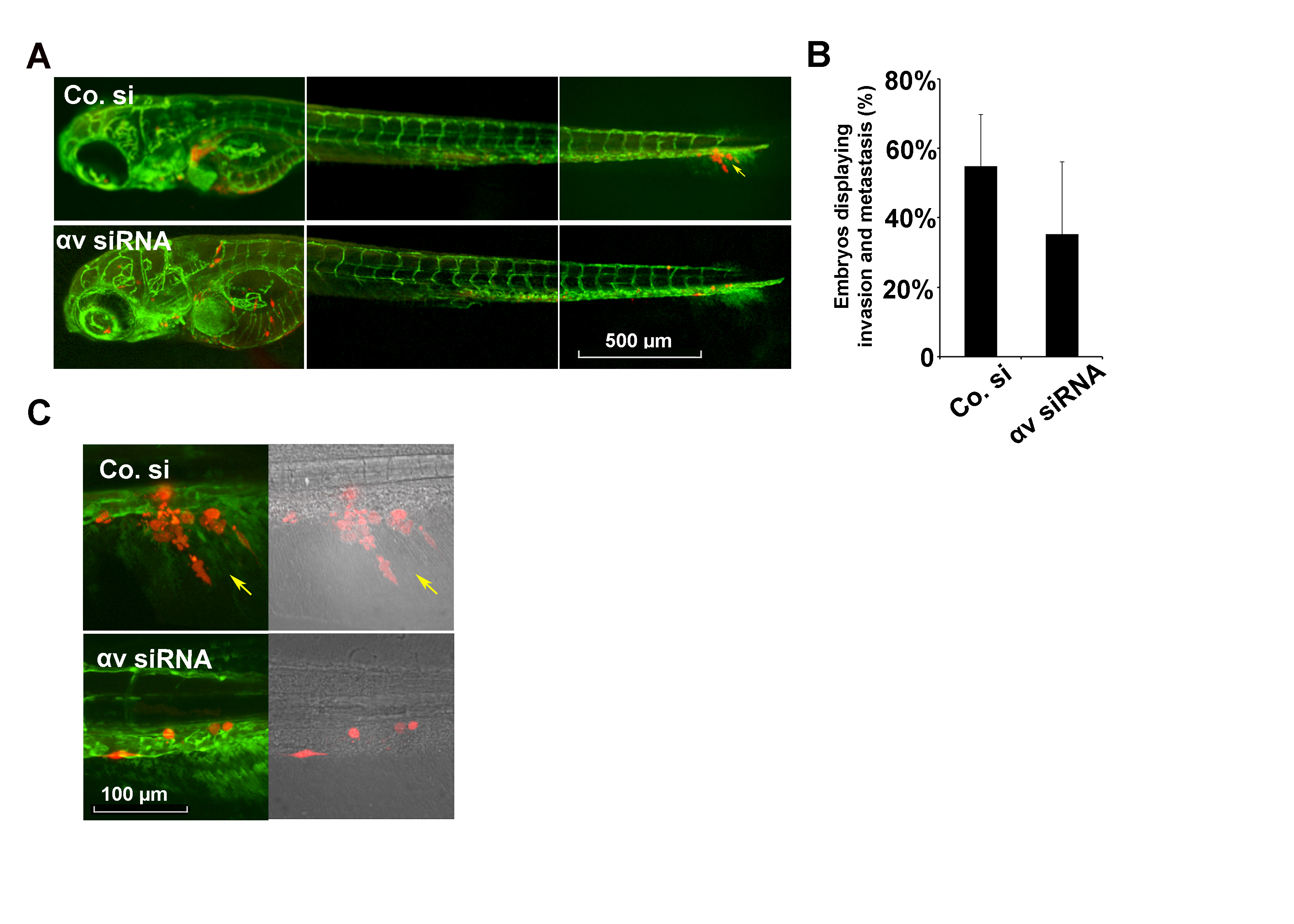

Supplement: Additional file 2: Figure S2. — Inhibition of tumor progression in zebrafish by siRNA-mediated knockdown of αv integrin. (A) fli1:GFP Casper zebrafish were injected with mCherry-labeled MDA-MB-231 cells transfected with control siRNA or αv integrin knockdown siRNA. Confocal images were photographed at 6 days post implantation (dpi). Arrows indicate invasive tumor cells, scale bar: 500 μm. (B) Percentage of embryos displaying invasion and metastasis at day 6 post-injection. Data are representative of two independent experiments (each, n >50). C. High-resolution images of the posterior tail to visualize single metastatic tumor cells (upper panel, fluorescence; lower panel, transmitted). Arrows indicate invasive tumor cells, scale bar: 100 μm. [file 13058_2015_537_MOESM2_ESM.jpeg]

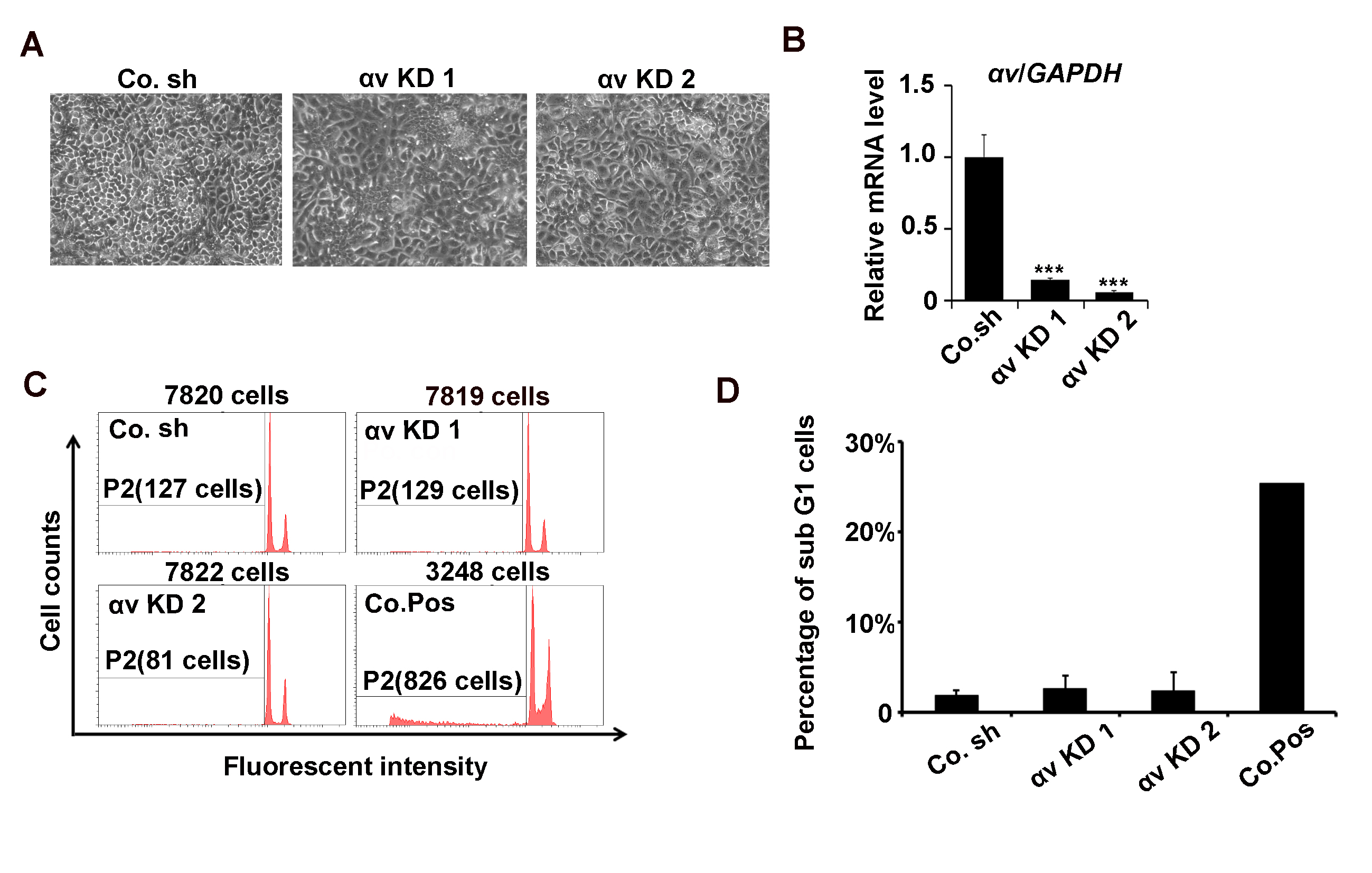

Supplement: Additional file 3: Figure S3. — Stable knockdown of αv integrin in MCF10A-M4 cells. (A) Representative images of MCF10A-M4 cells infected with lentivirus expressing control-shRNA or αv integrin knockdown shRNAs. (B) mRNA level of αv integrin in control and αv integrin knockdown MCF10A-M4 cells detected by quantitative PCR. (C) Representative Fluorescence-activated cell sorting (FACS) profiles of propidium iodine-stained control M4 cells, αv integrin KD 1 and 2 MCF10A-M4 cells and as positive apoptotic control doxorubicin treated MCF10A-M4 cells (Co.Pos). P2: subG1 cells. (D) Percentage of sub G1 cells calculated by FACS. Average ± standard deviation of 3 independent experiments). [file 13058_2015_537_MOESM3_ESM.jpeg]

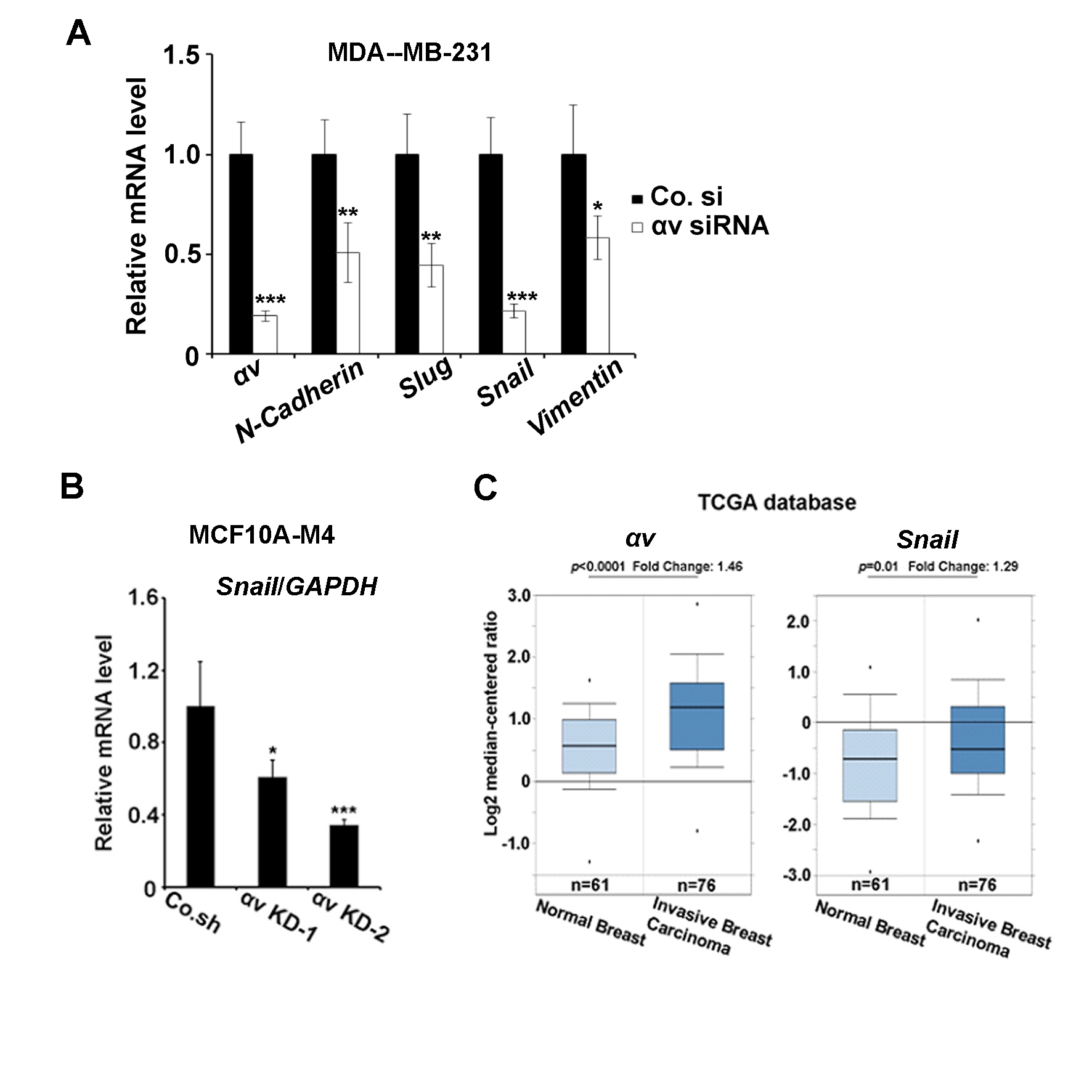

Supplement: Additional file 4: Figure S4. — Effects of αv integrin on mesenchymal markers. A. mRNA expression of mesenchymal makers in control siRNA or αv integrin siRNA transfected MDA-MB-231 cells. The mRNA levels of αv integrin, N-Cadherin, Slug, Snail and Vimentin are normalized to GAPDH expression. P-values were calculated by the two-sided Student t-test. (B) Quantitative PCR analysis of Snail mRNA in control and αv integrin knockdown MDA-MB-231 cells. The RNA levels (± SD) of Snail are normalized to GAPDH expression. (C) Oncomine™ box plots of αv integrin and Snail expression levels in normal breast and invasive breast carcinoma [38]. [file 13058_2015_537_MOESM4_ESM.jpeg]

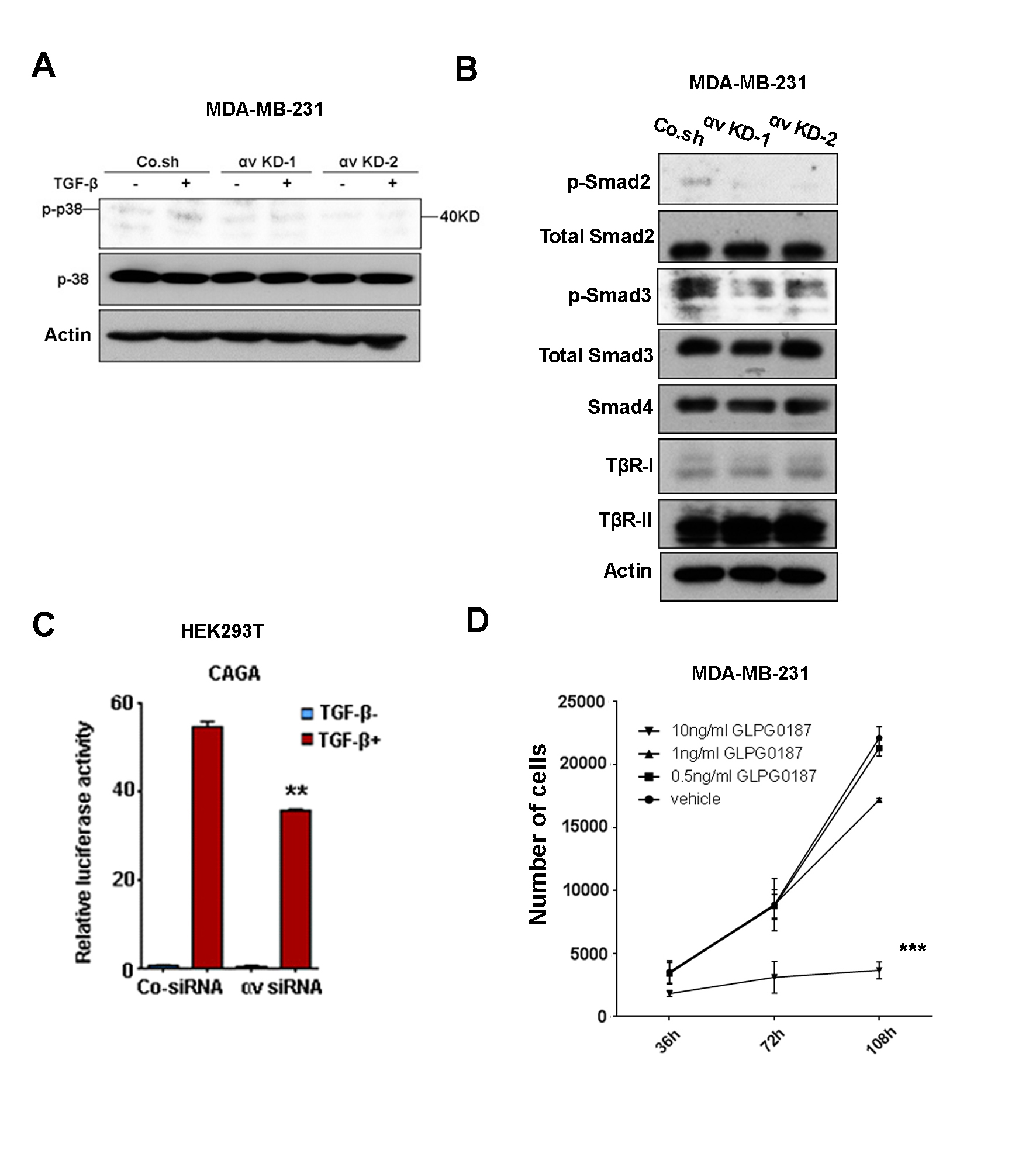

Supplement: Additional file 5: Figure S5. — Effect of genetic depletion of αv integrin on expression of transforming growth factor (TGF)-β pathway components and effect of GLPG0187 on cell proliferation/viability in MDA-MB-231 cells. (A) Western blot analysis of total and phosphorylated p38 in control and αv integrin knockdown MDA-MB-231 cells with or without 5 ng/ml TGF-β for 2 h. (B) Immunoblot analysis of phosphorylated Smad2 and 3, total Smad2 and 3, Smad4, TGF-β receptors in MDA-MB-231 αv integrin knockdown cells. (C) Analysis of the TGF-β-induced Smad3 transcriptional response: 293 T cells were transfected with the CAGA12-Luc transcriptional reporter, control siRNA or αv siRNA. Cells were treated with or without 5 ng/ml TGF-β for 16 h. (D) Proliferation curves of MDA-MB-231 cells upon treatment with vehicle, 0.5, 1 or 10 ng/ml GLPG0187 for 36, 72 and 108 h. [file 13058_2015_537_MOESM5_ESM.jpeg]
